# Effect of Steam Explosion on Structural Characteristics of β−Conglycinin and Morphology, Chemical Compositions of Soybean Meal

**DOI:** 10.3389/fnut.2022.896664

**Published:** 2022-06-02

**Authors:** Feng Kong, Qinghua Zeng, Yue Li, Xingfeng Guo

**Affiliations:** College of Agronomy, Liaocheng University, Liaocheng, China

**Keywords:** steam explosion, β-conglycinin, soybean meal, structural characteristic, chemical composition

## Abstract

In this study, steam explosion was applied as a means to degrade β-conglycinin. We investigated changes in morphology, the chemical composition of soybean meal, and the structural characteristics of β-conglycinin. The results showed that steam explosion at 0.7 MPa for 8 min could effectively decrease the β-conglycinin content of soybean meal while the histamine content was not increased. The structural characteristics of soybean meal proteins were analyzed by sodium dodecyl sulfate-polyacrylamide gel electrophoresis (SDS-PAGE), Fourier transform infrared spectroscopy (FTIR), circular dichroism (CD), and X-ray diffraction (XRD). Steam explosion caused the degradation of high weight proteins and reduced the band density of α’, α, and β subunits in β-conglycinin. The micro-surface of soybean meal seemed to be in the cracked or puffed stage and the color became brown or dark after steam explosion. Steam explosion facilitated the dissolution of water-extractable arabinoxylans, which are 4.81 fold higher than that of native soybean meal. Phytic acid was exposed to the hydrothermal environment of the steam explosion process and consequently degraded by 12.95–24.69%. The 2,2-diphenyl-1-picrylhydrazyl (DPPH) radical scavenging activity of soybean meal extract was gradually increased from 20.70 to 33.71% with the rising of treated pressure from 0.3 to 0.7 MPa, which was 1.11–1.81 fold of native extract. The steam explosion may be a new modification technology that could decrease antigenicity, and steam-exploded soybean meal (0.7 MPa, 8 min) with lower β-conglycinin and phytic acid content that could be widely used in food products.

## Introduction

Soybean meal is a major by-product of soybean oil production that contains large amounts of protein rich in essential amino acids (1, 2). Soybean meal is accessible and inexpensive, contains a high nutritional value, and is popular in the feed industry. However, soybean meal has been limited in food applications because of its practical disadvantages and undesirable allergens. The spatial structure of the subunits of soybean protein, such as β-conglycinin, has immunogenicity spatial epitopes that can induce a human allergic reaction. The dietary fibers and pectic substances in the cell wall form a complex matrix, which agglomerates with the proteins of soybean meal (3, 4). Soybean protein and bioactive components availability are reduced by cell walls, the anti-degradation barrier, consisting of insoluble and complicated carbohydrates (5). The previous studies evaluated the effects of different methods for pretreatment of soybean meal, such as fermentation by microorganisms (6), heat treatment (7), and enzymatic hydrolysis (8). However, these treatments face problems, such as the length of time they take, which limit their application. Therefore, it is necessary to find an effective method to break the complex structure of cell walls in soybean meal and decrease the β-conglycinin content before its utilization.

Steam explosion as an efficient pretreatment method that has been widely used to pretreat lignocellulosic materials (9, 10). High-pressure saturated steam was used to pretreat material in a closed reactor and released the pressure instantaneously (9). The steam explosion broke the cell wall structure of wheat bran, and led to the increase of soluble dietary fiber content and reduced lipase activity and phytic acid content (9–12). Steam explosion hydrolyzed the glycosidic bonds and decreased the molecular weight in potato starch (13). The steam explosion destroyed the physical bonds between and within the protein molecules of camellia seed cake, and decreased the number of large aggregates (14). The steam explosion enhanced the release of phenolic compounds and the antioxidant capacity of soybean seed coats (15). It is a novel hydrothermal processing technology in the food industry with high-efficiency and low-energy consumption, which was usually employed in high-fiber or high-protein materials (9, 16, 17).

The effect of steam explosion on the structural characteristics of β-conglycinin and morphology, and chemical compositions of soybean meal were investigated in this study. The β-conglycinin and histamine content, sodium dodecyl sulfate-polyacrylamide gel electrophoresis (SDS-PAGE), Fourier transform infrared spectroscopy (FTIR), X-ray diffraction (XRD), and circular dichroism (CD) of soybean meal proteins were analyzed. The effects of steam explosion on micro-surface, color, chemical composition, and 2,2-diphenyl-1-picrylhydrazyl (DPPH) radical scavenging activity of soybean meal were investigated. In this study, the steam explosion was expected to act as a potential desensitization strategy of legumes for developing hypoallergenic products.

## Materials and Methods

### Sample Preparation

Soybean meal was purchased from Jinshiliu Horticulture Co., Ltd. (Jiangsu, China). Steam explosion pretreatment of soybean meal was performed in self-designed equipment which mainly consisted of a steam generator (WY19, Big Soldier Food Machinery, Henan, China) and a reactor chamber (WY19, Big Soldier Food Machinery, Henan, China). Soybean meal was loaded into a reactor chamber from the feed valve, closed feed valve, and charged high-pressure saturated steam to the reactor chamber. Then, the reactor pressure was maintained at 0.3–0.7 MPa for 8 min, respectively. Afterward, the soybean meal samples were collected and dried in the oven at 60°C for 12 h. Dried soybean meal (100 g) was ground for 2 min in the ZT-150 grinder (Yongkang Zhanfan Industry and Trade Co., Ltd., Zhejiang, China).

The protein of the soybean meal was extracted using the method of Yang et al. (18). Soybean meal flour (0.05 g) was dispersed in 0.05 mol/L of Tris–HCl buffer (1 ml, pH 8.2), then placed in an ultrasonic bath for 15 min and incubated at 40°C. After extraction, the samples were centrifuged at 6000 rpm for 40 min by a centrifuge. The supernatant was dialyzed against distilled water with a dialysis membrane (MWCO, 3500 Da) at 4°C for 24 h, and then freeze-dried.

### Morphological Analysis of Soybean Meal

The scanning electronic micrographs (SEM) of soybean meal were assessed by a microscope (Thermo Scientific, Helios G4 CX) at a voltage of 1.00 kV. The native and steam-exploded soybean meal samples were dried in a vacuum freeze-dryer (FD-1C-50, Biocool, Beijing, China) before measurement.

### Chemical Composition Analysis of Soybean Meal

Total arabinoxylans and water-extractable arabinoxylans content were determined using the dual-wavelength colorimetry method of Hashimoto et al. (19). The protein of soybean meal was analyzed using the AACC method 46–08. The phytic acid content of soybean meal powder was determined using the method of Chen et al. (20). Soybean meal powder was mixed with 0.01 mol/L hydrochloric solution at a solid-to-liquid ratio of 1:10 (w/v, g/ml), then the extract was mixed with ferric chloride and sulfosalicylic acid, and the mixture was measured at 500 nm.

### Characterization of Allergen β-Conglycinin of Soybean Meal Powder

#### Determination of β-Conglycinin and Histamine Content of Soybean Meal Powder

The β-conglycinin and histamine content in soybean meal powder were determined in duplicate using the plant β-conglycinin ELISA Kit and Plant Histamine (HIS) ELISA Kit (ChunDuBio, Hubei, China), respectively. The level of β-conglycinin and histamine in samples was determined by the double antibody sandwich method. The color change is measured by Rayto RT-6100 microplate reader at a wavelength of 450 nm.

#### Sodium Dodecyl Sulfate-Polyacrylamide Gel Electrophoresis Analysis of Soybean Meal Proteins

The subunit profiles of protein were analyzed by using SDS-PAGE according to Laemmli (21) with modification. The unstained protein molecular weight marker from 14.4 to 116.0 kDa was used as the standard. Briefly, 1.5 mg/ml of sample was denatured at 100°C for 5 min with an equal volume of 0.125 M Tris–HCl buffer solution (pH 6.8) containing 20% glycerol, 4% SDS, 0.01% bromophenol blue, and 10% β-mercaptoethanol. The amount of protein dispersion transferred to each well was about 20 μl.

#### Infrared Spectrum Information of Soybean Meal Proteins

The variation in the structure of protein was evaluated by the Fourier transform infrared spectroscopy (FTIR) using an FTIR-Nicolet 460 E.S.P. spectrometer (Nicolet, United States), 32 scans at a resolution of 4 cm^–1^ according to the method of Zhao et al. (22). Disks were prepared by mixing 2 mg of dried soybean meal samples with 200 mg of KBr. The region of amide III of proteins was curve-fitted with Gaussian bands using PeakFit v4.12 software (SeaSolve Software Inc.) (23).

#### Circular Dichroism Spectrum Measurements of Soybean Meal Proteins

Circular dichroism spectroscopy of soybean meal proteins was performed with a JASCO Corp., J-810, Rev. 1.00 (Jasco, Germany) according to Pi et al. (24). Samples (0.6 mg/ml) were scanned in the far UV range from 250 to 190 nm at room temperature. The resulted spectra represented an average of three consecutive scans.

#### X-Ray Diffraction of Soybean Meal Proteins

The structures of protein samples were measured using a diffractometer (Rigaku, SmartLab 9 kW) according to Sharif et al. (25). The protein samples were scanned from 5 to 80° with a scanning speed of 0.02°/s.

### Color Measurements of Soybean Meal Powder

The color profiles of native and steam-exploded brown rice powders were measured with a chromameter (Minolta CR-10, Japan). L* means lightness of the powder, a* indicates green or red-purple color, and b* indicates yellow or blue color (18). Chroma (26), total color difference (ΔE) (26), and browning index (27) were calculated using L*, a*, and b* values.

### 2,2-Diphenyl-1-Picrylhydrazyl Radical Scavenging Activity of Soybean Meal Extract

The DPPH radical scavenging activity of soybean meal extract was determined according to the method of Kong et al. (9). Soybean meal powder (1 g) was dispersed in 80% of methanol solution (v/v, 10 ml) and centrifuged at 12,000 rpm for 10 min; the supernatant was made in 10 ml. The supernatant (0.1 ml) was added with 6.25 × 10^–5^ mol/L of DPPH methanol solution (2 ml) and reacted in the dark for 30 min, and then the absorbance was measured at 517 nm.

### Statistical Analysis

Three replicate tests were carried out and the average values were reported. Experimental data were processed by one-way analysis of variance using IBM SPSS Statistics 20 (IBM, NY, United States) with Duncan’s multiple range test (*p* < 0.05). The results were reported as mean ± standard deviation.

## Results and Discussion

### Microstructural Characterization of Soybean Meal

**FIGURE 1:**
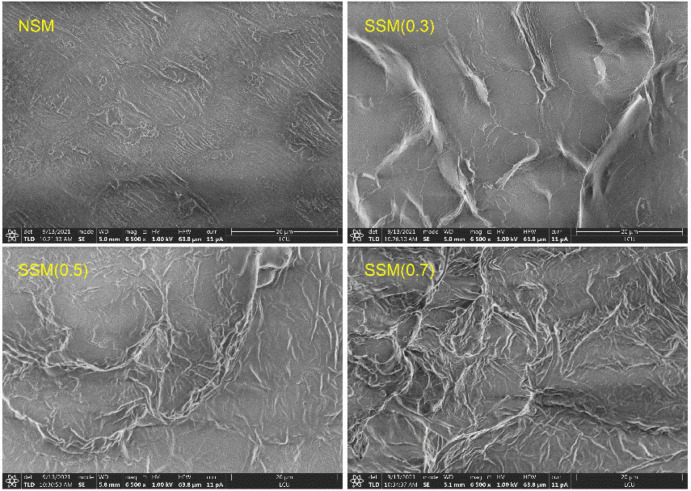
The scanning electronic micrograph (SEM) images of soybean meal. NSM, native soybean meal; SSM, steam-exploded soybean meal, SSM (0.3), SSM (0.5), and SSM (0.7) indicated steam explosion pressure were 0.3, 0.5, and 0.7 MPa, respectively.

The microstructural changes of steam-exploded soybean meal were investigated in Figure 1. The SEM clearly showed that the micro-surface of the native soybean meal was more intact and compact. After the steam explosion process, some palpable changes in microstructural characterization were observed: the micro-surface of soybean meal became cracked and rough. Hydrothermal atmosphere, strong explosion, and sheared force of steam explosion treatment could soften and hydrolyze the lignin and hemicellulose, which lead to the broken dense structure of soybean meal and the melted state of soybean meal micro-surface (28). The instant explosion further destroyed the morphology and structure of the soybean meal, and exposed internal substances, and might be conducive to the separation of cell wall matrix and protein, which could improve the accessibility and degradation efficiency of proteins from soybean meal.

### Chemical Composition Analysis of Soybean Meal Powder

Arabinoxylans, water-extractable arabinoxylans, protein, and the phytic acid content of soybean meal powder are outlined in Table 1. Results showed the increased water-extractable arabinoxylans and decreased phytic acid content and no apparent changes of protein in steam-exploded soybean meal. Dietary fiber, including arabinoxylans, is the major constituent in the cell wall and provides a variety of health benefits (29). Water-extractable arabinoxylans content could be used as an important indicator to evaluate the effect of different treatments on cell wall degradation (30). The degree of arabinoxylans cross-linking is known to be a factor controlling the toughness of plant cell walls (31). The steam explosion could promote the conversion of arabinoxylans to water-extractable arabinoxylans; it broke the crystalline structure of the cell wall. When the pressure of the steam explosion increased from 0.3 to 0.7 MPa, the water-extractable arabinoxylans content reached the maximum value of 17.59 mg/100 g, which were 5.81 fold higher than that of native soybean meal. The steam explosion was an efficient tool for breaking the cell wall of plant-based feedstock under the double action of saturated steam at a high-temperature and high-pressure. The steam was an excellent plasticizer for softening dietary fibers and it promoted the physical tear of insoluble arabinoxylans converted to soluble arabinoxylans at sudden decompression (32). Then hydrolysis reactions were carried out under mild acidic conditions which come from a decrease of water pK_w_ at high temperatures and the release of organic acids from steam penetrated feedstock (33). The effects were partly responsible for the steam explosion that facilitated the major destruction of arabinoxylans in the cell wall of soybean meal. The mechanical action of the steam explosion caused the exposure of internal substances in soybean meal and improved the accessibility of proteins from agglomerates in the soybean meal cell walls, which was beneficial to thermal and mechanical denature proteins. Phytic acid was the anti-nutritional factor that could chelate metal ions which significantly lowered ions absorption. It is widely found in the aleurone layer of cereals bran and cotyledons (beans and oilseeds) (34). It will reduce the bioavailability and nutrient value of foods without appropriate processing (12). The phytic acid contents of soybean meal powder treated by the steam explosion were significantly decreased (*p* < 0.05) as compared with the native soybean meal. The steam explosion combined chemical and thermal effects, which formed a mild acidic environment by auto-hydrolysis of hemicellulose. The phytic acid might be degraded by exposure to the high temperature and acidic conditions of steam explosion from soybean meal (35, 36).

**TABLE 1:** Chemical composition of soybean meal powder.

Samples	AX (%)	WEAX (%)	Protein (%)	Phytic acid (mg/g)
NSM	28.52 ± 0.12^a^	3.03 ± 0.32^d^	45.33 ± 1.41^a^	27.10 ± 0.97^a^
SSM(0.3)	22.66 ± 0.33^b^	6.19 ± 0.37^c^	45.83 ± 1.84^a^	23.59 ± 0.06^b^
SSM(0.5)	23.09 ± 0.22^b^	10.06 ± 0.65^b^	43.86 ± 5.64^a^	20.43 ± 0.87^c^
SSM(0.7)	22.77 ± 0.37^b^	17.59 ± 0.22^a^	45.94 ± 0.44^a^	20.41 ± 0.09^c^

*NSM, native soybean meal; SSM, steam-exploded soybean meal, SSM (0.3), SSM (0.5), and SSM (0.7) indicated steam explosion pressure were 0.3, 0.5, and 0.7 MPa, respectively. AX, arabinoxylans, WEAX, water-extractable arabinoxylans (means in each column followed by different letters are significantly different, p < 0.05).*

### Effect of Steam Explosion on Allergen β-Conglycinin

#### β-Conglycinin and Histamine Content of Soybean Meal Powder

**FIGURE 2:**
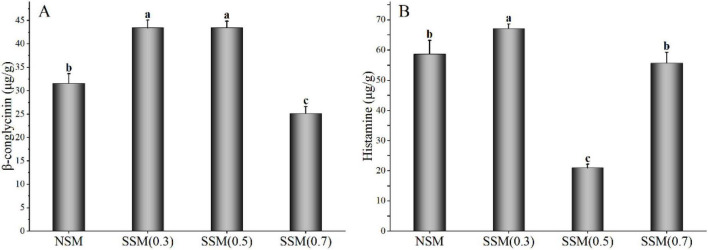
The β-conglycinin and histamine content of soybean meal powder. **(A)** β-Conglycinin content. **(B)** Histamine content. NSM, native soybean meal; SSM, steam-exploded soybean meal, SSM (0.3), SSM (0.5), and SSM (0.7) indicated steam explosion pressure were 0.3, 0.5, and 0.7 MPa, respectively (means that do not share a letter are significantly different, *p* < 0.05).

The β-conglycinin was the main antigenic protein in soybean meal (37). After the treatment of steam explosion, the β-conglycinin content of soybean meal powder changed to varying degrees (Figure 2). Among them, the β-conglycinin contents of steam explosion treatment at 0.3 and 0.5 MPa were significantly higher than that of native soybean meal, while 0.7 MPa for 8 min was lower (*p* < 0.05). Soybean protein availability was reduced by the structural barrier of soybean outer layers, which consisted of complex insoluble carbohydrates (5). The steam explosion (0.3 and 0.5 MPa) broke the cell wall structure, which exposed protein might increase β-conglycinin extractability of soybean meal powder. Under the double action of high-temperature and high-pressure from saturated steam, steam explosion (0.7 MPa, 8 min) modified the exposed proteins from soybean meal. Poisoning with histamine can produce symptoms, such as urticarial, and the histamine content was regarded as a criterion of food quality (38). Compared with a native sample, the histamine content significantly increased by the steam explosion at 0.3 MPa and decreased by the steam explosion at 0.5 MPa (*p* < 0.05). At 0.7 MPa for 8 min, the steam explosion significantly decreased β-conglycinin content (*p* < 0.05) but did not increase histamine content (*p* > 0.05) of soybean meal compared with that of native soybean meal. However, the mechanism of histamine content changes induced by the steam explosion is unclear. Future study is still needed to investigate the influence and mechanism of different steam explosion conditions on allergens content, and self-designed experimental apparatus for the steam explosion is needed continuous improvement. The characterization of β-conglycinin of protein extract affected by the steam explosion was discussed in the following experiments.

#### Sodium Dodecyl Sulfate-Polyacrylamide Gel Electrophoresis Profile of Soybean Meal Proteins

**FIGURE 3:**
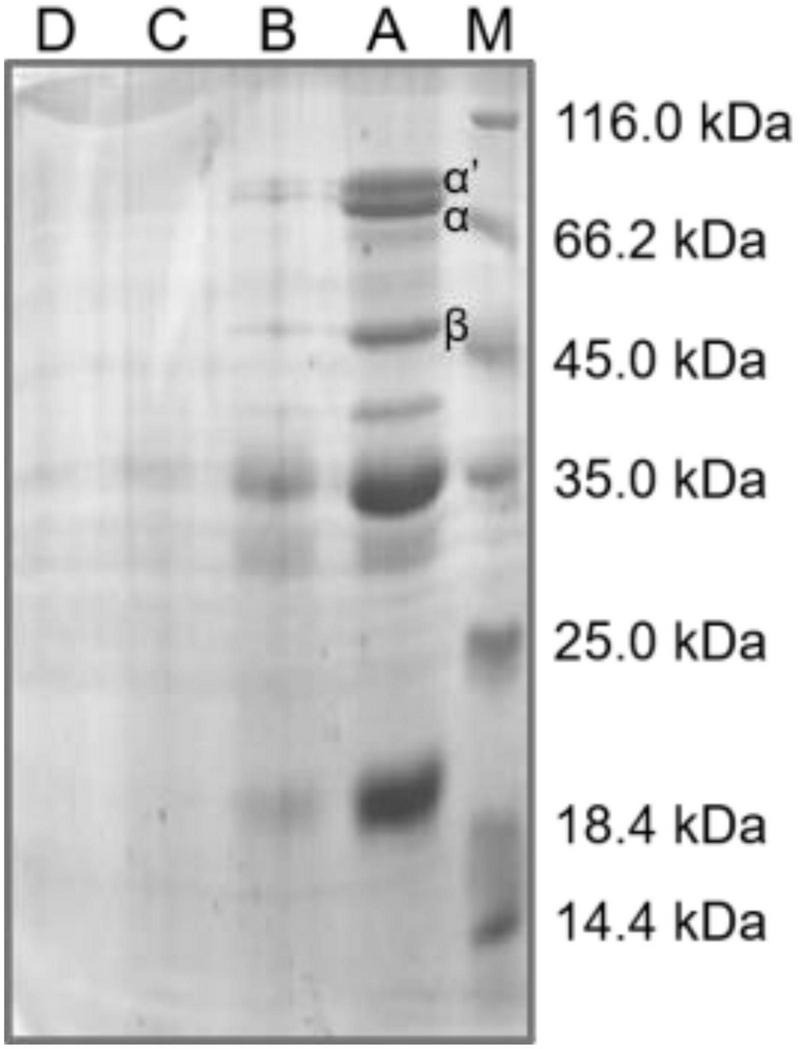
The sodium dodecyl sulfate-polyacrylamide gel electrophoresis (SDS-PAGE) profile of soybean meal protein. M, standard protein markers; A, native soybean meal; B, C, and D indicated soybean meal treated by steam explosion at 0.3, 0.5, and 0.7 MPa, respectively.

The SDS-PAGE was usually utilized to assess the subunit profiles and binding type of protein aggregation in soybean meal (39). Glycinin and β-conglycinin were the main antigenic proteins in soybean meal; glycinin was composed of 37–42 kDa and 16–21 kDa; and β-conglycinin mainly consisted of α’ (78 kDa), α (72 kDa), and β (52 kDa) (18). The α subunit of β-conglycinin was the most allergenic, causing allergic reactions in mice, piglets, and calves. The protein patterns of soybean meal are shown in Figure 3. The molecular distribution of all the soybean meal proteins could be divided into five regions of 66.2–116.0, 45.0–66.2, 35.0–45.0, 25.0–35.0, and 18.4–25.0 kDa. Compared with native soybean meal, the steam explosion caused significant changes in protein profiles; the intensity of the electrophoresis bands for steam-exploded soybean meal protein was lower than that for the native soybean meal protein. When the steam pressure was at 0.3 MPa, the relatively thick protein bands near 52, 72, and 78 kDa became thinner compared with that of the native sample. The protein profile became unstable above 0.5 MPa, and at 0.7 MPa, virtually all protein bands disappeared. The mechanical energy and acid environment generated by the steam explosion probably destroyed the disulfide-bond of the subunit structure in soybean meal protein, dissociated protein aggregation, and reduced the band density of the α′, α, and β subunits of β-conglycinin. Soybean meal was fermented by *Bacillus subtilis* for 24 h in the degradation of soybean meal antigenic proteins, the band density of α′, α, and β subunits in β-conglycinin was also reduced (18). Soybean meal was hydrolyzed with alcalase for 10 min; the α′, α, and β subunits of β-conglycinin were decreased by the SDS-PAGE pattern (8). Compared with fermentation and enzymolysis, the steam explosion saved processing time and cost, and might be conductive to improving industrial production efficiency.

#### Fourier-Transform Infrared Spectroscopy of Soybean Meal Proteins

**FIGURE 4:**
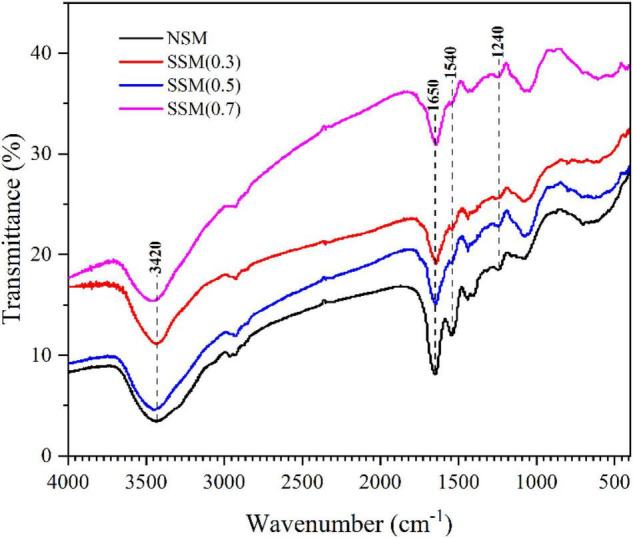
The infrared spectrum information of soybean meal protein. NSM, native soybean meal; SSM, steam-exploded soybean meal, SSM (0.3), SSM (0.5), and SSM (0.7) indicated steam explosion pressure were 0.3, 0.5, and 0.7 MPa, respectively.

In this study fourier transform infrared spectroscopy was performed to investigate the structural changes of protein in the steam-exploded soybean meal. As shown in Figure 4, the FTIR peaks displayed a marked difference between the native soybean meal and steam-exploded soybean meal. The absorption peaks at around 3400, 1700–1600, 1580–1480, and 1330–1220 cm^–1^ referred to the characteristic peaks of amide A (N–H stretching), amide I (*C* = O stretching), amide II (N–H bending vibrations or C–N stretching vibration), and amide III (C–O and C–N stretching, *O* = C–N and N-H bending) (23, 40–42). The FTIR spectrum of protein obtained after the steam explosion revealed changes in the characteristic peaks of the amides. The high temperature and acidic environment during the cooking stage, the instantaneous decompression action during the explosion stage of the steam explosion process was effective in triggering the denaturation of proteins (43), which significantly reduced the intensities of the absorption peaks of amides I and amide II, and broke down the long-chain protein in the short (22), compared to native soybean meal protein.

**FIGURE 5:**
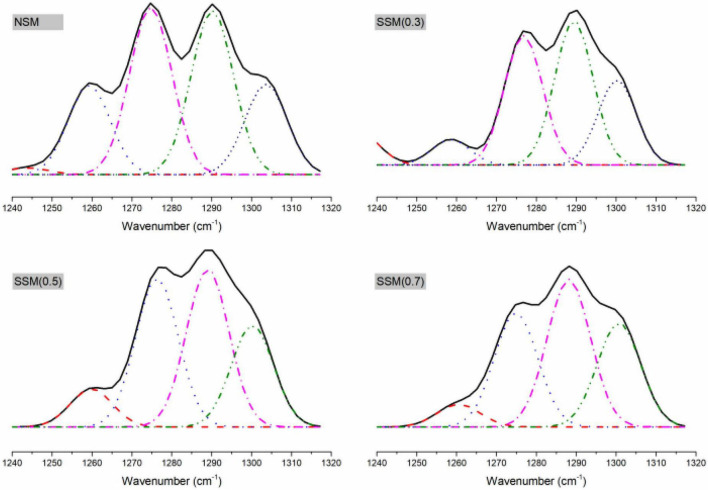
The Fourier transform infrared spectroscopy (FTIR) peak resolution of amide III spectral regions, 1320–1240 cm^–1^. NSM, native soybean meal; SSM, steam-exploded soybean meal, SSM (0.3), SSM (0.5), and SSM (0.7) indicated steam explosion pressure were 0.3, 0.5, and 0.7 MPa, respectively.

The deconvolution of the amide III regions was more accurate for analyzing of secondary structure in the protein, the microstructural components could be estimated according to α-helix, β-turn, random coil, and β-sheet with 1330–1295 cm^–1^, 1295–1270 cm^–1^, 1270–1250 cm^–1^, and 1250–1220 cm^–1^, respectively (44, 45). Figure 5 indicated that the steam explosion could greatly change the secondary structure of the protein. The native soybean meal protein contained 1.30% β-sheet, 64.06% β-turn, 17.20% disordered regions, and 17.44% α-helix. The steam-exploded soybean meal protein (0.7 MPa, 8 min) contains 67.44% β-turn, 5.82% disordered regions, and 26.74% α-helix. The antigenicity of β-conglycinin might be related to the content of β-turn and random coils (46). This result revealed that β-sheet content could be decreased in the steam-exploded protein while β-turn content increased. The changes in secondary structure indicated dissolution and regeneration induced the denaturation of protein structure by steam explosion (14).

#### Circular Dichroism Spectrum Analysis of Soybean Meal Proteins

**FIGURE 6:**
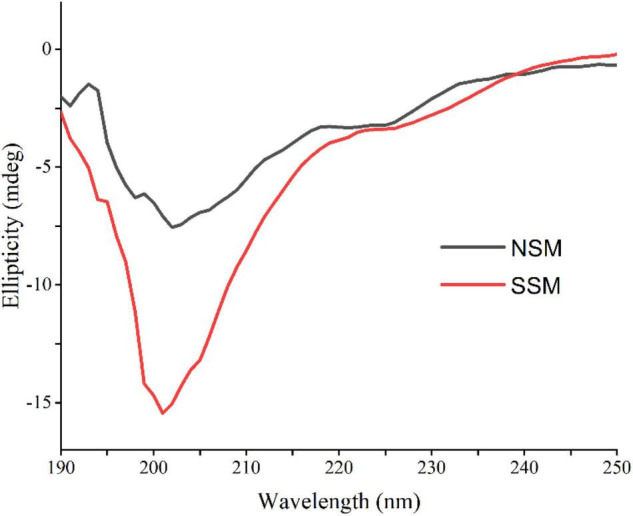
Circular dichroism spectra of native and steam-exploded soybean meal proteins. NSM, native soybean meal; SSM, steam-exploded soybean meal.

As shown in Figure 6, steam explosion (0.7 MPa, 5 min) altered the secondary structures of soybean meal proteins markedly. The intensity of the band varied between native and steam-exploded samples for the 0.6 mg protein per ml buffer solution, suggested that secondary structural shift might be occurred. The single positive peaks at around 192 and 199 nm were indicative of the α-helix and β-sheet, the predominant random coil structure from proteins in CD spectra were the negative troughs at around 205 nm, which indicated steam-exploded sample showed a gross loss in both apparent α-helix and β-sheet contents (47). Steam explosion increased the protein flexibility while decreasing the protein stability (48).

#### X-Ray Diffraction Analysis of Soybean Meal Proteins

**FIGURE 7:**
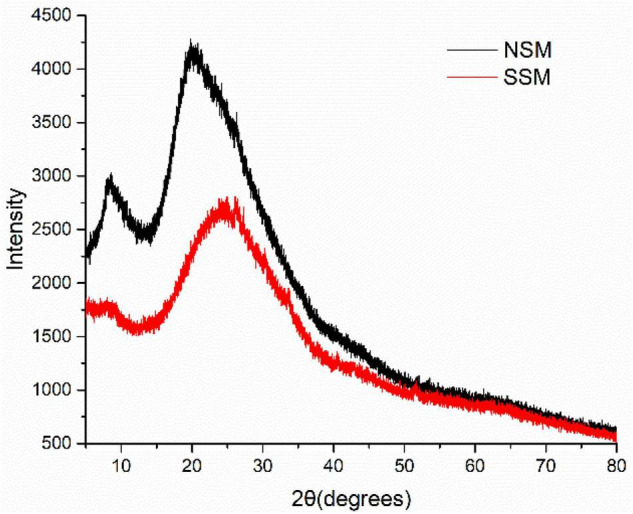
The X-ray diffraction (XRD) patterns of the proteins from native- and steam-exploded soybean meal. NSM, native soybean meal; SSM, steam-exploded soybean meal.

The crystalline characteristics of the native- and steam-exploded (0.7 MPa, 8 min) soybean meal proteins were studied by XRD (Figure 7). Proteins obtained from native soybean meal showed a high-crystal peak at the 2θ values of about 10 and 20°. The peak angles of α-helix structure from proteins in X-ray patterns were at 2θ around 10° and the peak angles of the β-sheet structure were at 2θ around 20° (49). The relative crystallinity associated with the crystalline structures of proteins decreased and changed by steam explosion through the double action of steam temperature and pressure. The intensities of the native- and steam-exploded soybean meal proteins at 2θ about 10 and 20°, showed that the percentage of α-helix and β-sheet in the proteins obtained from the steam-exploded soybean meal was lower compared to that obtained from the native soybean meal. XRD data showed that the intensities of β-conglycinin at 2θ about 10° were significantly decreased by steam explosion (50). This suggested that steam explosion could disrupt the advanced spatial structure of the soybean meal proteins at the gas explosion stage through mechanical action.

### Color Measurements of Soybean Meal Powder

**FIGURE 8:**
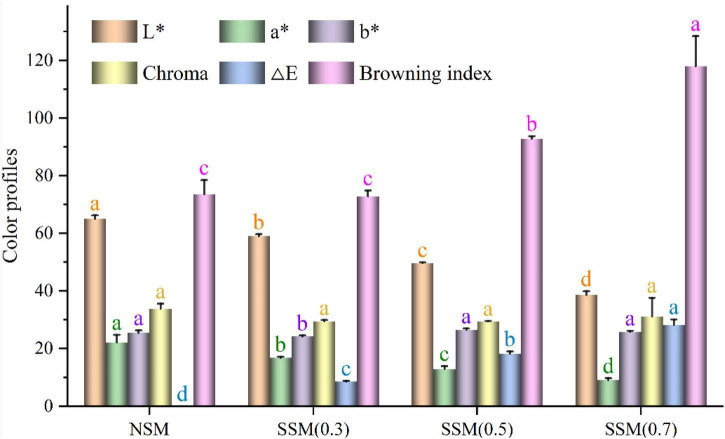
Color measurements of soybean meal powder. NSM, native soybean meal; SSM, steam-exploded soybean meal, SSM (0.3), SSM (0.5), and SSM (0.7) indicated the steam explosion pressure were 0.3, 0.5, and 0.7 MPa, respectively (means that do not share a letter are significantly different, *p* < 0.05).

The steam explosion-treated soybean meal samples had lower L* and a* values, but exhibited no change in b* values without SSM (0.3) as compared to NSM (L* = 65.07, a* = 22.17, b* = 25.47) (Figure 8). Color parameters indicated that steam explosion treatment of soybean meal yielded a darker color (lower L* and a* values). A similar result was obtained by Zhao et al. (51), wherein steam-exploded wheat bran exhibited a significantly darker color than raw wheat bran. When the pressure increased from 0.3 to 0.7 MPa, the lightness decreased from 59.00 to 38.60, which was lower than that of native soybean meal (65.07). The total color difference (ΔE) of steam-exploded soybean meal powders varied from 8.60 to 28.20, which was typically used to evaluate the degree of the total differences between steam-exploded and native soybean meal powders. The chroma values of steam-exploded powders indicated the purity or saturation, and showed no significant variation compared to native powder (26). No change was found in chroma between native- and steam-exploded soybean meal powders (*p* > 0.05), which indicated the stability of yellow color in soybean meal powders. The browning index was an important parameter in processes that represented the purity of the brown color (27). Browning index changed between 72.77 and 118.02 in the steam explosion process, which was significantly higher (*p* < 0.05) than that of native powder (73.55), except for steam explosion at 0.3 MPa (72.77) (*p* > 0.05). These results indicated that the steam explosion strongly affected the color quality of brown rice and produced more brown compound(s) with the extending of treatment pressure. Several other investigations have reported similar observations (12, 51). There was a remarkable negative correlation (*p* < 0.01) between L* and ΔE (*r* = –0.989), L* and browning index (*r* = –0.950), a* and b* (*r* = –0.785), a* and ΔE (*r* = –0.961), a* and browning index (*r* = –0.791), while L* and a* (*r* = 0.937), ΔE and browning index (*r* = 0.919) showed significant positive relationship (*p* < 0.01). The undesired Maillard browning reaction, caramelization, and oxidation product formation could be responsible for the decrease of lightness in steam-exploded soybean meal compared to the native soybean meal (12, 43). The fermentation-assisted enzymatic-treated soybean meal also had a lower L* (63.10–69.21) value compared to native soybean meal (L* = 81.39) (18). Misfortune might be a blessing in disguise; the color change could be advantageous in cookies (52).

### Antioxidant Property of Soybean Meal Extract

**FIGURE 9:**
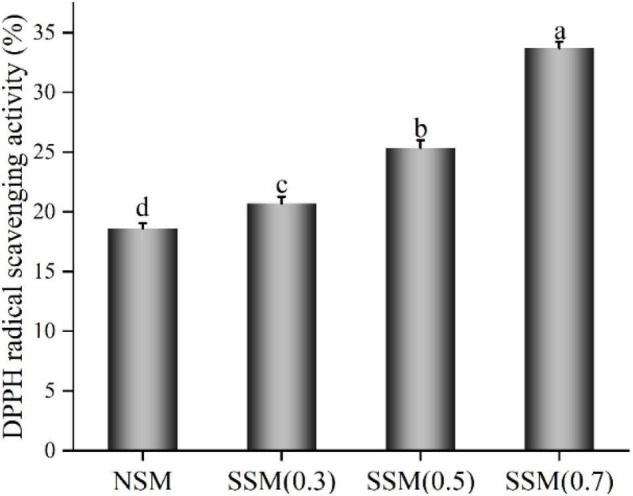
Antioxidant property of soybean meal extract. NSM, native soybean meal; SSM, steam-exploded soybean meal, SSM (0.3), SSM (0.5), and SSM (0.7) indicated steam explosion pressure were 0.3, 0.5, and 0.7 MPa, respectively (means that do not share a letter are significantly different, *p* < 0.05).

The DPPH radical scavenging activity of soybean meal powder is listed in Figure 9. The DPPH radical scavenging activity of soybean meal extract showed the main differences among them. When the pressure of steam explosion increased from 0.3 to 0.7 MPa, the DPPH radical scavenging activity reached the maximum value of 33.71%, which was 1.81 fold that of native soybean meal. Steam explosion (0.7 MPa, 8 min) broke the cell wall of soybean meal, thus increasing the water-extractable arabinoxylans and phytochemicals content, degraded protein by thermal and mechanical actions and could have contributed in part to the increase in DPPH radical scavenging activity. Steam-exploded soybean meal powder (0.7 MPa, 8 min) with the lowest β-conglycinin, phytic acid content, and highest DPPH radical scavenging activity could be used as an ideal functional ingredient in flour products.

## Conclusion

This study demonstrated that the allergen β-conglycinin content and structural characteristic in soybean meal could be decreased and modified by steam explosion at 0.7 MPa for 8 min while histamine content was not increased. The decrease of β-conglycinin content could be interpreted in the hydrothermal effect of steam explosion and Maillard reactions that occurred between reducing sugar and proteins. Steam-exploded soybean meal showed higher water-extractable arabinoxylans, DPPH radical scavenging activity. In addition, steam explosion significantly reduced the phytic acid content in soybean meal powder under thermal and acidic actions, which may improve the nutritional property and potential utilization of soybean meal. Therefore, the steam explosion could have good prospects in legume utilization. In future studies we will aim to improve the effect of a self-designed steam explosion device, and investigate the immunologic property of soybean meal allergens.

## Data Availability Statement

The original contributions presented in the study are included in the article/supplementary material, further inquiries can be directed to the corresponding author.

## Author Contributions

FK and XG: conceptualization, resources, and supervision. FK, YL, and QZ: methodology, validation, and investigation. FK: software, formal analysis, data curation, writing-original draft preparation, writing-review and editing, project administration, and funding acquisition. QZ and FK: visualization. All authors have read and agreed to the published version of the manuscript.

## Conflict of Interest

The authors declare that the research was conducted in the absence of any commercial or financial relationships that could be construed as a potential conflict of interest.

## Publisher’s Note

All claims expressed in this article are solely those of the authors and do not necessarily represent those of their affiliated organizations, or those of the publisher, the editors and the reviewers. Any product that may be evaluated in this article, or claim that may be made by its manufacturer, is not guaranteed or endorsed by the publisher.
